# Reflex(ions)

**Published:** 1888-01

**Authors:** C. M. Wright

**Affiliations:** Cincinnati


### Reflex-(ions).

BY C. M. WRIGHT, D.D.S., CINCINNATI.

[Read before the Ohio State Dental Society, Springfield, Ohio, October, 1887.]

There seems to be a growing tendency on the part of some of the practitioners of a sister specialty in medicine, to trespass upon our special field. This has been observed occasionally for some years, and the articles in the New York *Medical Record* a year or two ago, were only more pronounced expressions of the idea entertained by a few, at least, of the members of this sister specialty. I refer to the disposition of certain occulists, who, in forming diagnoses of the diseases of the eye, attempt to investigate the condition of the teeth of their patients, justly acknowledging to themselves, the influence that abnormal irritation in this part, might have, reflexly, upon the tissues of the eye. The dental profession has always admitted, even before any definite knowledge of the subject had been gained by the experiments in this direction by modern physiologists, the possibility of a very intimate connection between the teeth and the eyes through the afferent and efferent nerves supplied to both.

The *vox populi* had long ago recognized the relation of the teeth to near and distant parts of the body, and the superior cuspids, as we know by their popular name and by common consent of the laity, are believed to be intimately connected with the eyes. We know by this same evidence, that the inferior cuspids are believed to be intimately associated with the stomach. Gastric troubles may be and are no doubt induced, reflexly, by disease of the inferior cuspids. This is not ridiculous. It is like many another medical fact, now abundantly proven by late scientific research, which had long been foreshadowed, as it were, by the broad proverbs of the people.

It is not ridiculous, when we think of the interdependence of the tissues to the body, each upon all the others. When we study the physiology of any, even the most simple and common reflex act, and follow the impression that is made upon the end of a sensory nerve, along the track of the sensory nerve to the centre, and back again to the various muscular tissues of the body, and then try to note and measure its effect upon the heart, the lungs, the vessels of the entire body, as well as upon the infinite number of muscle cells of the particular part especially, and visibly affected, and toward which the impulse had been directed, we then begin to appreciate the wonderful interdependence of all the tissues of a complex organism.

To be able to see clearly all that is going on in my body at this moment, from the effects of this written page held in my hand and photographed by the rays of light reflected from it, on my retina, where it becomes an irritant and the cause of an impulse and final nervous explosion affecting the digestive, vascular, respiratory, and other structures of my body, would be to understand all the phenomena of reflex activities. If we could possess this power of perception of physiological conditions, how easy it would be to trace out variations of the same as developed in pathological conditions! We could then estimate exactly the value as an injurious irritant of every phase of dental abnormality. We would know just how much extra work a "sensitive cavity" in a tooth would impose upon the heart and lungs and vessels and other tissues of the entire body; just how much, more or less, of oxygen must be taken in; just how much, more or less, of carbonic acid gas must be given out; just how much, more or less, the secretive and excretive functions of the tissues must be exercised to balance and settle up with the great mogul, Nutrition. But we don't know these things yet, gentlemen. We are like children on the shore of a great ocean; we hear the roar of the waves; we see the ships sail by; we know even the geography of the sea; we have gathered shells and anemonies and read the story of Jonah and the whale, but we cannot begin to estimate the effects of the stone cast by our hand into the water. The ripples of circles are believed to go on widening and widening until they reach the uttermost shores, and are then reflected back again, and so on for ever and ever. So it is with nerve wavelets in our organisms—every passing breath that causes a sensation, vibrating through our sensitive nerves—returning after many days and years, and passing on into infinity. There is just so much in this subject of reflex action.

Now, why did I begin this paper by saying that occulists trespassed upon our field? Simply because opthalmology has made no greater strides toward unraveling these mysteries than has odontology, and occulists are as much like children on the shore of the ocean of reflex activities as are dentists, and practically while occulists have devoted their microscopic attention to the eye, dentists have done the same to the teeth—and the occulist is no better fitted by this sort of study to diagnosticate lesions of the dental organs, than is the dentist to diagnosticate diseases of the eye.

When an occulist sends his patient to a dentist with the request that this or that tooth should be extracted, he is behaving in precisely the same manner and with no higher intelligence than would the dentist if he should send his patient to the occulist and request the enucleation of the right eye, because of a lesion of a right cusped tooth pulp; consultation and full explanations on both sides would be fair and honorable and profitable in either case.

A gentleman recently returned from the eye clinique of the Paris hospital said: "When a patient appears at the clinique for treatment, in Paris, the first thing we do is to examine his teeth, and have all stumps and roots and dead teeth extracted, then we begin on his eyes." Gentlemen, this is a broader view of a specialty practice than we have been in the habit of taking, why should we not emulate it, and as Tomes has ingeniously shown that the teeth are dermal appendages, why would it not be good for us in our clinics and private practices as well, to greet a patient who applies to us for professional attention to the dental apparatus, with, " My dear madam take off your stockings and let me look at your feet; have you corns? Go to the chiropodist and have them extracted and then I can treat your masticators." (?) This would not be so very far out of the way, we must admit, for we know that reflexly an irritated corn or bunion affects and causes profound impressions on the nerve centres which discharge volumes of energy through remote nerve channels communicating with the muscles of the brow, cheeks, lips, larynx, lungs, and a whole host of other muscles of the organism. Why should we not infer then that the teeth are included in the list?

I have been encouraged in expressing these opinions by the reports from the Ninth International Medical Congress which give the inaugural address of Nathan Smith Davis, M.D., L.L. D., President of the Congress. In this address the President says: "Indeed the most defective and embarrassing features in the science and art of medicine, at this time, is the rapid accumulation of facts furnished by the vast numbers of individual workers, each pushing investigations in some special direction without concert with his fellows, and without any adequate conception of the coincident lines of observation necessary to enable him to see the true bearing of the facts he evolves. Hence he is constantly mistaking mere coincidences for the relation of cause and effect, and the pages of our medical literature are being filled with hastily formed conclusions and rules of practice from inadequate date."

I have also felt at liberty to make these remarks and to call opthalmology a sister specialty, after reading an editorial from Dr. J. Taft, in the October number of the DENTAL REGISTER, in which he says: "Dentistry, as a specialty of the healing art, has, within the past few months, been *officially* placed side by side with other departments of medical practice."

This, it seems to me gives us the privilege of inaugurating a family quarrel.

